# Supplementary material for: Genetic Diversity in Phytoplasmas from X-Disease Group Based in Analysis of idpA and imp Genes
Source: Microorganisms. 2025 May 21;13(5):1170. doi: 10.3390/microorganisms13051170 (PMC12114192; doi:10.3390/microorganisms13051170)
Supplement: Supplementary file 1 [file microorganisms-13-01170-s001.zip › microorganisms-3581236-supplementary.pdf]

*Article*

# Genetic Diversity in Phytoplasmas From X-Disease Group Based in Analysis of *idpA* and *imp* Genes

**Florencia Ivette Alessio [1,2], Vanina Ayeln Bongiorno [1,2], Carmine Marcone [3], Luis Rogelio Conci [1,2] and Franco Daniel Fernandez [1,2,*]**

## Supplementary Material

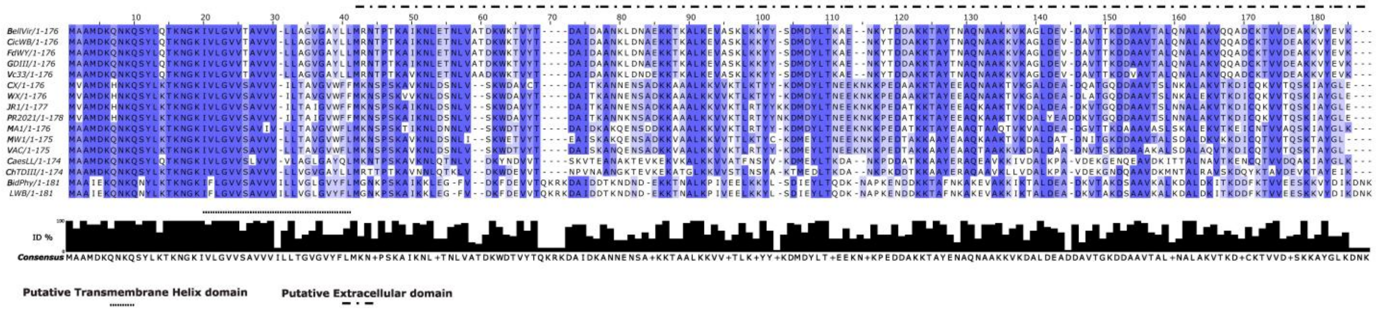


**Figure S1.** Multiple sequence alignment of imp proteins from representative strains of X-disease (16SrIII) group. Conserved residues are indicated by a gradient of blue intensity. Predicted transmembrane helix domains are marked with solid lines (---), while putative hydrophilic extracellular domains are indicated with dash-dot lines (-.-).

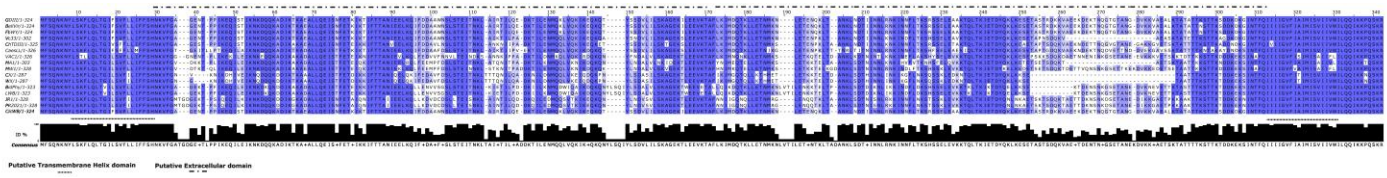

**Figure S2.** Multiple sequence alignment of idpA proteins from representative strains of X-disease (16SrIII) group. Conserved residues are indicated by a gradient of blue intensity. Predicted transmembrane helix domains are marked with solid lines (---), while putative hydrophilic extracellular domains are indicated with dash-dot lines (-.-).

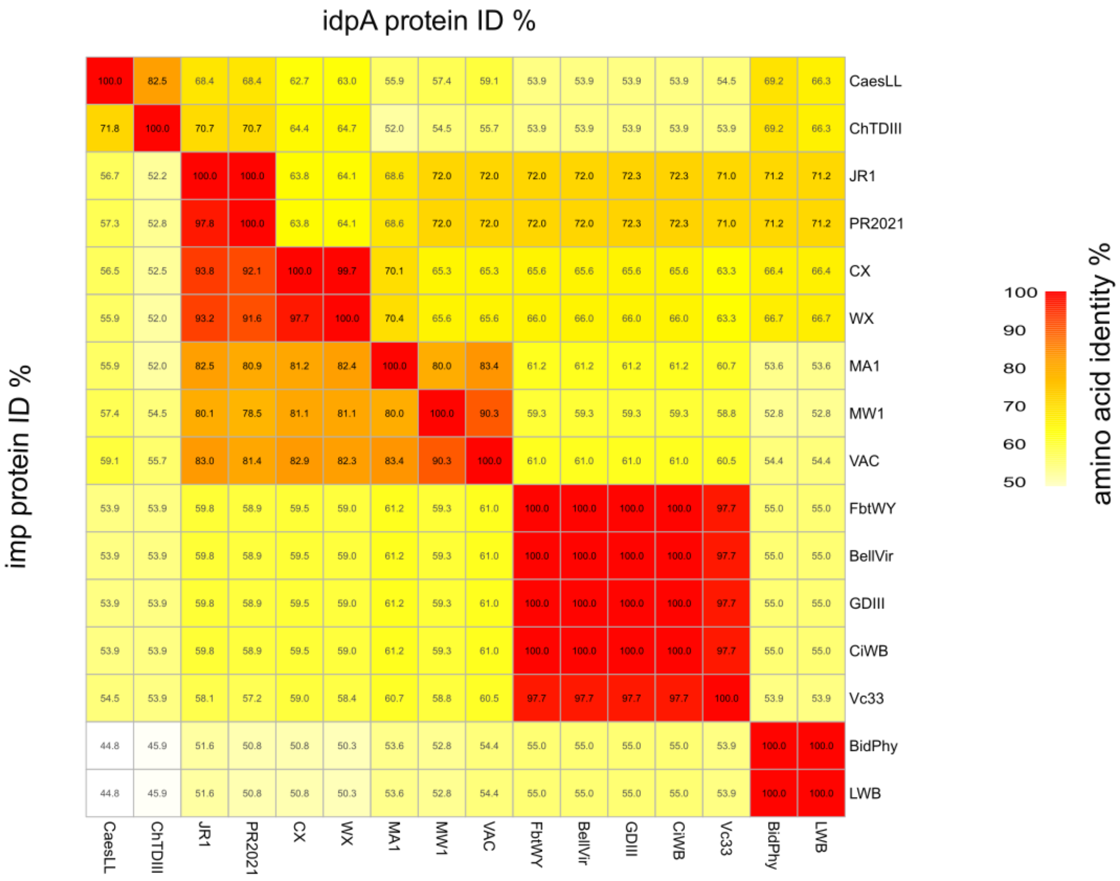


**Figure S3.** A pairwise sequence identity matrix (%) comparing the two proteins, imp (lower diagonal) and idpA (upper diagonal), across the same bacterial isolates. The values represent percentage identity, with colors (in the original heatmap) ranging from cool tones (low identity, e.g., 40–60%) to warm tones (high identity, e.g., 80–100%). The diagonal (100.0) serves as a self-identity reference.

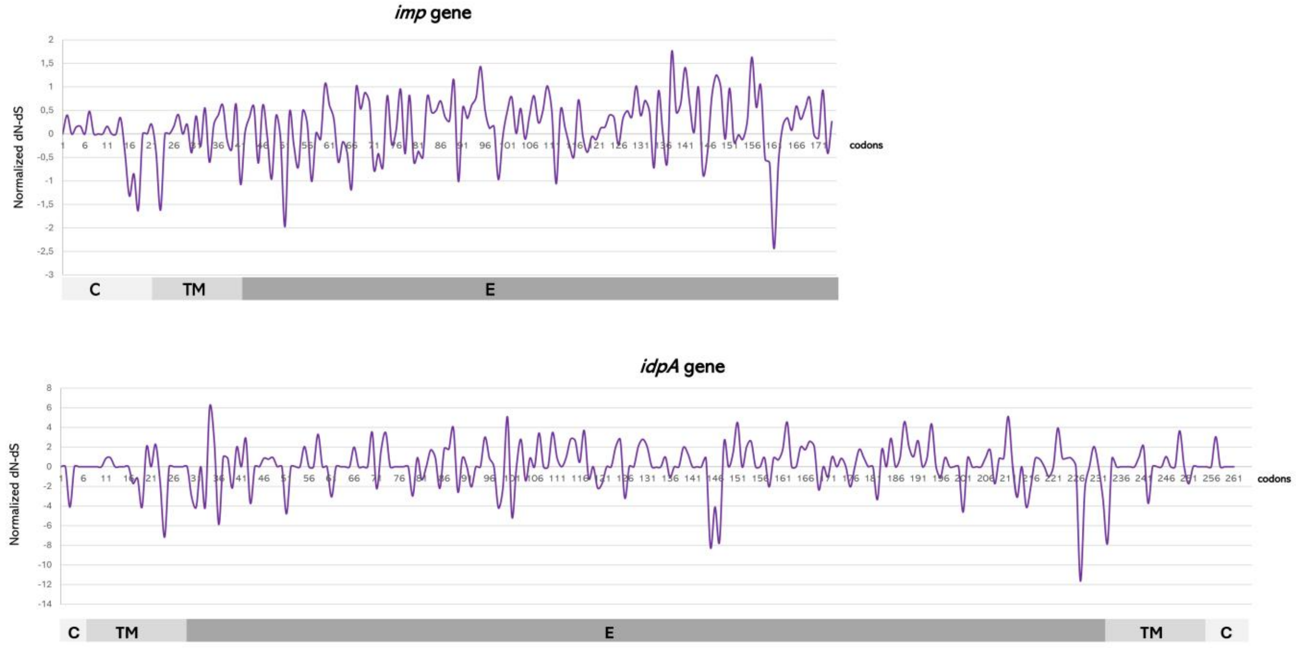


**Figure S4.** The normalized dN/dS values across codon sites in the imp and idpA proteins. The plot illustrates site-specific selection pressures, with the normalized dN/dS values (y-axis) plotted against the codon positions (x-axis). The domains are annotated: C (cytoplasmic), TM (putative transmembrane helix), and E (extracellular hydrophilic). The values are derived from maximum likelihood ancestral reconstructions (16 sequences; gaps excluded).

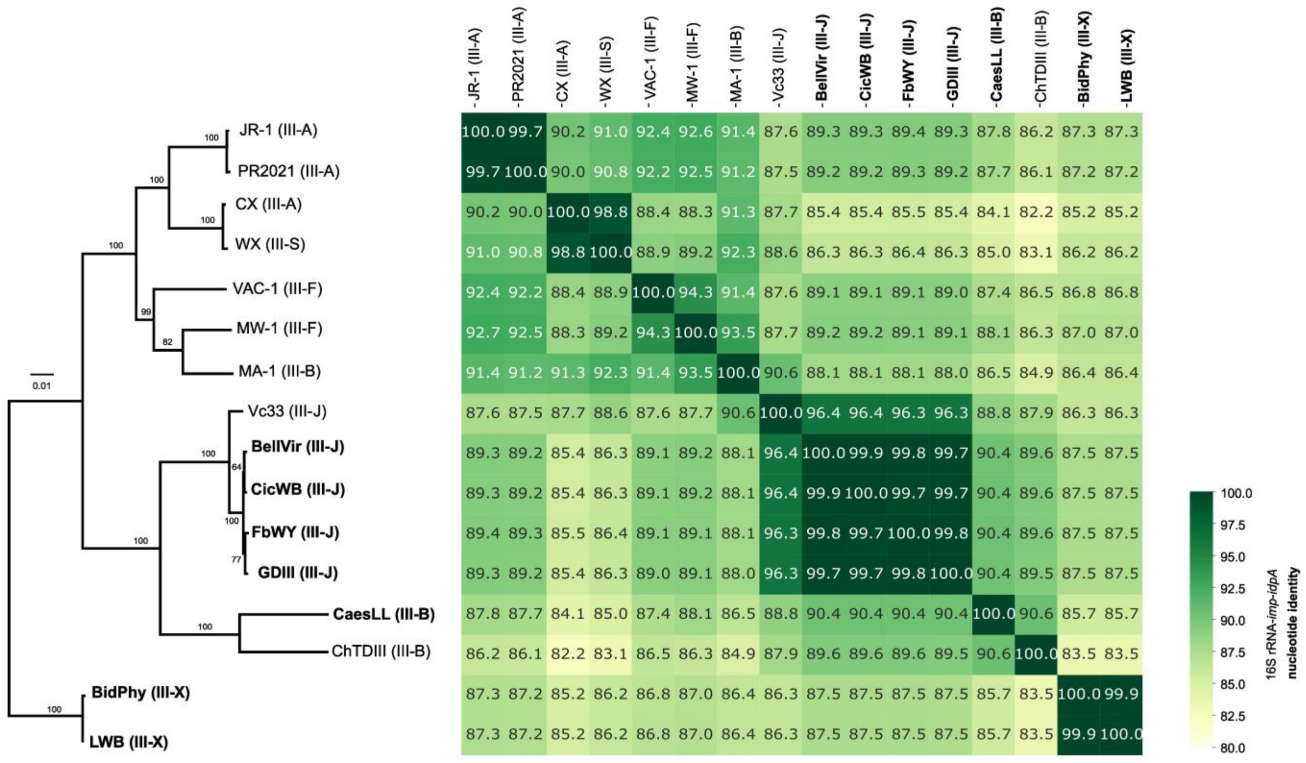


**Figure S5.** A phylogenetic tree and pairwise nucleotide identity matrix based on the concatenated sequences (16S rRNA, imp, and idpA genes; 2794 bp) of the 16SrIII group phytoplasmas. The heatmap shows the pairwise percentage of nucleotide identity, while the dendrogram represents

the phylogenetic relationships inferred from the concatenated alignment using the ML method. Color gradients indicate identity values ranging from 80% (light yellow) to 100% (dark green). The bootstrap support values (1000 replicates) are indicated at the main nodes. In bold are the sequences obtained in this work.
